# Unveiling Novel Metalloprotease Inhibitors Targeting Botulinum Neurotoxin Through Structure-Based Virtual Screening of Drug Libraries and Molecular Dynamics Simulations

**DOI:** 10.3390/ijms27188081

**Published:** 2026-09-11

**Authors:** Ridwan Sulaimon, Gurudeeban Selvaraj, Nora W. C. Chan, Anguang Hu, Gilles H. Peslherbe

**Affiliations:** 1 Centre for Research in Molecular Modeling (CERMM), Department of Chemistry and Biochemistry, Concordia University, Montreal, QC H4B 1R6, Canada; ridwan.sulaimon@mail.concordia.ca (R.S.); gurudeeban.selvaraj@avmc.edu.in (G.S.); 2 Suffield Research Centre (SRC), Defence Research and Development Canada, Government of Canada, Medicine Hat, AB T1A 8K6, Canada; nora.chan@forces.gc.ca (N.W.C.C.); anguang.hu@drdc-rddc.gc.ca (A.H.)

**Keywords:** *Clostridium*, botulinum neurotoxin, drug repurposing, hydroxamates, binding affinity, molecular docking, molecular dynamics simulations

## Abstract

Botulinum neurotoxin (BoNT) is one of the most lethal biological substances to humans, which inhibits acetylcholine release by the presynaptic nerve in neuromuscular junctions. Of the existing BoNT serotypes, BoNT serotype A (BoNT/A) is particularly potent, making it the primary focus in neurotoxin research. Its catalytic domain exhibits similar structural features and zinc-dependent activity as thermolysin, a key bacterial enzyme, which provides a foundation for designing antibacterial agents targeting related protease mechanisms. Repurposing of preapproved drugs or existing medications has recently proven an effective strategy to accelerate drug discovery. Accordingly, we employed drug-likeness screening, quantitative estimation of drug-likeness (QED), and molecular docking to screen about 9000 ligands from the FDA-preapproved drug library using the known crystal structures of the toxin’s light chain, and we identified potential inhibitors with the highest binding affinity. We further refined our selection using molecular dynamics simulations to investigate the stability of the receptor–ligand complexes. The binding mode analysis and binding free energies of the receptor–ligand complexes provide crucial information about the mechanism of action of our top-ranked potential inhibitors. Notably, 16 ligands exhibit a binding affinity greater (in magnitude) than that of the hydroxamate inhibitors that are co-crystallized in the X-ray structure. Most of these ligands contain fluorine, carboxylic and phosphate moieties as key functional groups that enhance their interactions with key residues of the BoNT active site. Our results suggest that dinoprost and 15 other clinically investigated ligands may serve as candidate scaffolds for further evaluation as potential BoNT/A LC inhibitors, pending experimental validation.

## 1. Introduction

Botulinum neurotoxins (BoNTs) are produced under anaerobic conditions by *Clostridium botulinum*. They are the most toxic substances known to human beings and are characterized by exceptional potency as food poisons, with a mouse LD_50_ of 0.1 ng/kg for type A [1,2]. Seven well-established BoNT serotypes, designated A through G, have been identified; of these, types A, B, E, and F cause human botulism, and type A is the most potent serotype [2,3]. Being exposed to BoNT/A can trigger botulism, an intense neuroparalytic illness defined by advancing flaccid paralysis. In extreme cases, paralysis of the respiratory muscles may lead to respiratory failure followed by death [4].

BoNTs are 150 kDa proteins formed by two chains connected through a disulfide bond. The heavy chain (HC, 100 kDa) serves an ancillary role by binding to the target nerve cells through its C-terminus and transferring the LC into the cytoplasm of the cell through its N-terminus [5]. The light chain (LC, 50 kDa) constitutes zinc-endopeptidases; they cleave one or more soluble N-ethylmaleimide sensitive factor attachment receptor (SNARE) protein complexes [6]. The BoNT/A LC selectively cleaves the neuronal protein SNAP-25, a SNARE complex component that mediates acetylcholine release from synaptic vesicles at neuromuscular junctions. Without functional SNAP-25, acetylcholine-dependent signaling to skeletal muscle stops entirely, which causes flaccid muscle paralysis.

BoNT/A LC belongs to the M27 family of zinc metallopeptidases in the MEROPS classification system [5,7]. Its catalytic domain contains the conserved HEXXH zinc-binding motif that defines many metalloproteases. This catalytic zinc ion is coordinated principally by His223, His227, and Glu262, while Glu224 participates in catalysis by activating a water molecule required for peptide-bond hydrolysis. Beyond the catalytic pocket, nearby residues help with recognizing the substrate and with how inhibitors attach. Metal coordination together with active-site flexibility and an extended substrate-binding cleft renders BoNT/A LC an intrinsically structurally sophisticated drug target [8,9,10].

At present, management of botulism relies on respiratory supportive care while antitoxin is given. Only equine antitoxin and BabyBIG^®^ are available as antitoxins; BabyBIG^®^ is derived from the blood of human donors vaccinated with a pentavalent (ABCDE) toxoid vaccine, and it is reserved for infant botulism. The period during which antitoxin can be administered is brief, and after the onset of symptoms, the antitoxin no longer provides benefit as it is unable to penetrate nerve cells. BoNTs cause muscle paralysis that lasts for several months [1,11] in part because the BoNT protease remains active in cells for an extended period, leading to prolonged dependence on ventilator support for months [12]. A bioterror attack can engender a public health crisis because prophylaxis and treatment measures are ineffective. Consequently, there is a need for therapeutic agents that can access intoxicated cells and directly inhibit the proteolytic activity of the intracellular light chain. Small-molecule inhibitors of BoNT/A LC could therefore support existing antitoxin strategies, extend the treatment window, and likely promote faster recovery from BoNT-driven paralysis [13,14,15]. The development of potent and selective BoNT/A LC inhibitors has nevertheless remained challenging.

Several inhibitors, including peptide-based compounds, hydroxamate-containing zinc chelators, quinolinol derivatives, and other nonpeptidic scaffolds [8], have been identified against BoNT/A LC. Hydroxamates and quinolinols have received particular attention because of their ability to coordinate the catalytic zinc ion. However, some reported quinolinol and hydroxymate inhibitors were either non-specific or toxic. Additionally, strong zinc chelation alone may not provide sufficient potency or selectivity because similar metal-binding motifs occur in other physiologically important metalloproteases. Effective inhibition therefore requires an appropriate balance between zinc coordination and complementary interactions with residues within the catalytic and substrate-recognition regions of BoNT/A LC. Active-site loop flexibility adds another layer of difficulty for inhibitor design, as the binding pocket geometry and accessibility can vary when ligand scaffolds differ [9,10].

The X-ray structures of BoNT/A metalloprotease complexed with different hydroxyamide substrate analogs, namely (2E)-3-(2,4-dichlorophenyl)-N-hydroxyacrylamide (GB4), 4-[bis(4-chlorobenzyl)amino]-N-hydroxybutanamide (QI1), and N-hydroxy-2-[(3S,5S,7S)-tricyclo[3.3.1.1~3,~]decc-1-yl] acetamide (AXM), were retrieved as 2IMA [9], 3QIY [10], and 4HEV [16], respectively, from the Research Collaboratory for Structural Bioinformatics-Protein Data Bank (RCSB-PDB). The inhibition constant (K_i_) of QI1, AXM, and GB4 against BoNT/A LC are 6.1 μM [10], 0.46 μM [16] and 0.30 μM [9], respectively. The moderate inhibitory activities of the co-crystallized ligands warrant a search for more potent BoNT/A metalloprotease inhibitors as therapeutic agents for botulism treatment.

Despite progress in structural characterization and inhibitor co-crystal design, the catalytic mechanism and intracellular trafficking of the BoNT/A light chain remain active topics of research. Recent high-resolution studies of related BoNT homologues provide additional structural templates that may inform inhibitor design beyond classical assemblies [17]. The mechanistic understanding of the BoNT domain architecture, its catalytic activity, and the host–cell interactions relevant for therapeutic targeting have been recently reviewed [18]. Beyond structure, emerging mechanistic studies highlight the complexity of BoNT/A intracellular trafficking and membrane association, factors that may influence effective inhibitor engagement in vivo [19]. While the canonical catalytic activity of the BoNT/A light chain centers on SNAP-25 cleavage, host phosphorylation networks and signaling pathways are increasingly being recognized as important biological contexts for toxin action and response, offering potential new avenues for therapeutic intervention and screening models [20].

Computer-aided drug discovery (CADD) tools have gained prominence as powerful tools in the identification of protein inhibitors and the study of protein drug and protein–protein interactions [21,22] as computational approaches provide time- and cost-effective means for identifying potential drug candidates [23]. Although previous studies have utilized CADD to explore some BoNT inhibitors [24], the search for clinically effective inhibitors is still ongoing. In the current study, structure-based virtual screening was conducted to identify candidate small-molecule inhibitors for the BoNT/A-LC protease. Experimentally characterized BoNT/A LC inhibitors were first compiled to evaluate the ability of the docking protocol to distinguish compounds with different levels of activity. The co-crystallized inhibitors GB4, QI1, and AXM were included as structural and energetic benchmarks. An initial library of FDA-approved and clinically investigated compounds was subsequently assembled from DrugBank [25] and curated using physicochemical and drug-likeness criteria [26]. The curated compounds were docked into the catalytic sites of the three BoNT/A LC structures using the Flexibility Induced Through Targeted Evolutionary Description algorithm (FITTED) implemented in the Molecular Forecaster platform. Subsequently, highly ranked complexes were investigated using molecular dynamics simulations to determine protein–ligand stability and the persistence of active-site interactions. Molecular mechanics and generalized Born surface area (MMGBSA) calculations were also conducted to estimate binding free energies and to select compounds with predicted affinities comparable to or exceeding those of the reference inhibitors. Through this integrated workflow, the study aimed to identify structurally diverse candidate inhibitors of the BoNT/A LC protease and characterize the molecular features responsible for their predicted binding and inhibitory potential.

## 2. Results

### 2.1. Binding Affinity Assessment and Validation of Reference Ligands

Experimental BoNT/A LC inhibitory data extracted from the BDB served as a basis for validation of ligand binding strengths evaluated by molecular docking. The reference ligand dataset includes 29 ligands with known half-maximal inhibitory concentration (IC_50_) values. These IC_50_ values were converted into relative empirical binding affinities (RBAs) to assess each ligand binding strength relative to that of the co-crystallized ligand QI1 (which was chosen as a reference due to its median FITTED score among the three reference ligands, Supplementary Information Table S1). Among the reference inhibitors, four (BDB11, BDB12, BDB13, and BDB14) exhibit weaker binding affinities than QI1, while the remaining inhibitors show moderate affinities on par with or exceeding that of the co-crystallized ligand AXM (Table S1).

**Figure 1:**
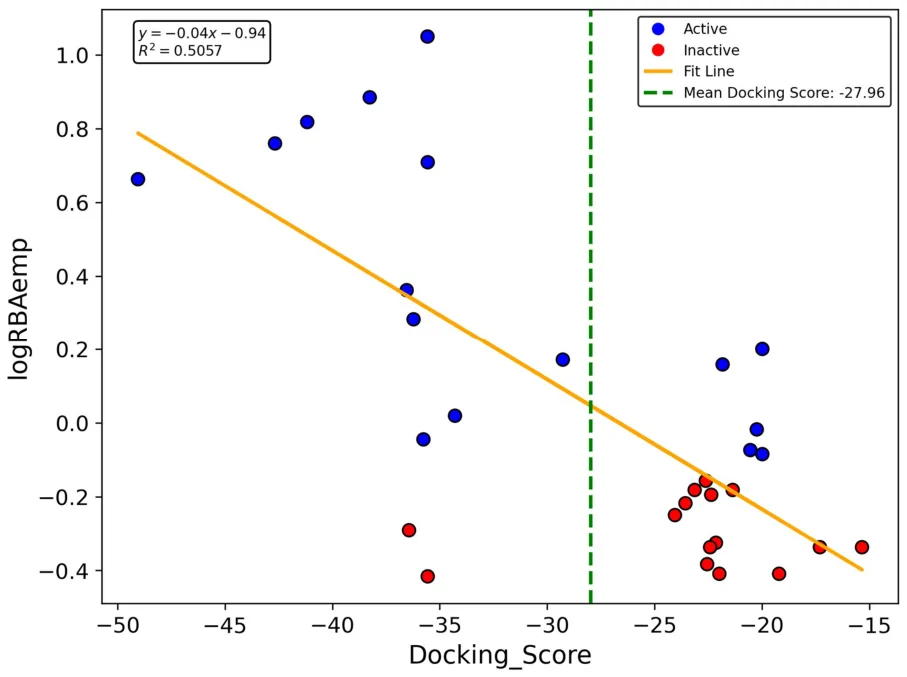
LogRBA vs. FITTED docking score for reference BDB ligands categorized as active (blue, IC_50_ ≤ 5 μM) and inactive (red, IC_50_ > 5 μM). The orange line represents a linear regression fit of the data and trend line. The green dashed line marks the arbitrary docking score cutoff of our binary model to discriminate between active and inactive inhibitors.

The molecular docking scores for the experimental BoNT/A LC inhibitors (also listed in Table S1) are consistent with relative binding affinities, as shown by the trend/weak correlation between logRBA and docking scores shown in Figure 1. It is remarkable that all reference inhibitors (active or inactive) have docking scores below −15, and we adopt a binary predictor model according to which a successfully docked ligand is classified as a potentially active BoNT-LC inhibitor if the docking score is less than −29. With this prescription, all experimentally inactive reference inhibitors but two are characterized as such on the basis of their docking scores. We note that the docking-score-based model will incorrectly classify five experimentally active compounds (with moderate activity) as inactive, but we do not deem this a serious limitation in our search for potent inhibitors with high activity. Attempts to construct a more quantitative predictive model for logRBA from computed molecular docking scores are mitigated by the small size of our reference dataset (e.g., a linear least-squares fit of logRBA vs. docking score has a poor regression coefficient of 0.5).

### 2.2. Molecular Docking-Based Virtual Screening

The resulting 7576 ligands that passed the Ro5 screening were taken as input for molecular docking-based virtual screening. The validity of virtual screening techniques and parameters was initially established by docking a collection of reference ligands from the Binding DB library, as well as the co-crystallized ligands QI1 (PDB ID 3QIY), AXM (PDB ID 4HEV), and GB4 (PDB ID 2IMA), to superimposed receptor structures. A negative FITTED score with a higher magnitude signifies a more favorable interaction between the ligand and BoNT/A. FITTED binding scores and binding energies for the reference compounds, including the redocked co-crystallized ligands QI1, AXM, and GB4, are collected in Table 1.

The validation process revealed that all three co-crystallized ligands involve H-bond interactions with residues Phe163, Glu224, and Tyr366; hydrophobic interactions with residues Phe194, Tyr366, and Phe369; and a coordinate bond with the zinc ion. These interactions align with previously reported experimental findings [10]. The binding interactions of the co-crystallized inhibitors with the BoNT/A metalloprotease are shown in Supplementary Information Figures S1–S3. Notably, X-ray crystal structures and docked structures exhibit significant overlap within similar regions inside the receptor. The docked pose of QI1 aligns with the X-ray crystal structure (PDB 3QIY) with a Root Mean Square Deviation (RMSD) value of 1.5 Å. Meanwhile, the docked pose of AXM and the X-ray structure (PDB 4HEV) exhibits an RMSD value of 2.1 Å, and the docked pose of GB4 and the X-ray structure (PDB 2IMA) an RMSD value of 1.1 Å. The docking protocol employed for the BoNT/A co-crystallized ligands was then applied to a previously screened dataset of 7576 ligands. Among these, 92 exhibit a FITTED score exceeding −50 (in magnitude). This benchmark was selected based on the best reference ligand score of −49 (Supplementary Information Table S2).

### 2.3. Molecular Dynamics Simulations

**Figure 2:**
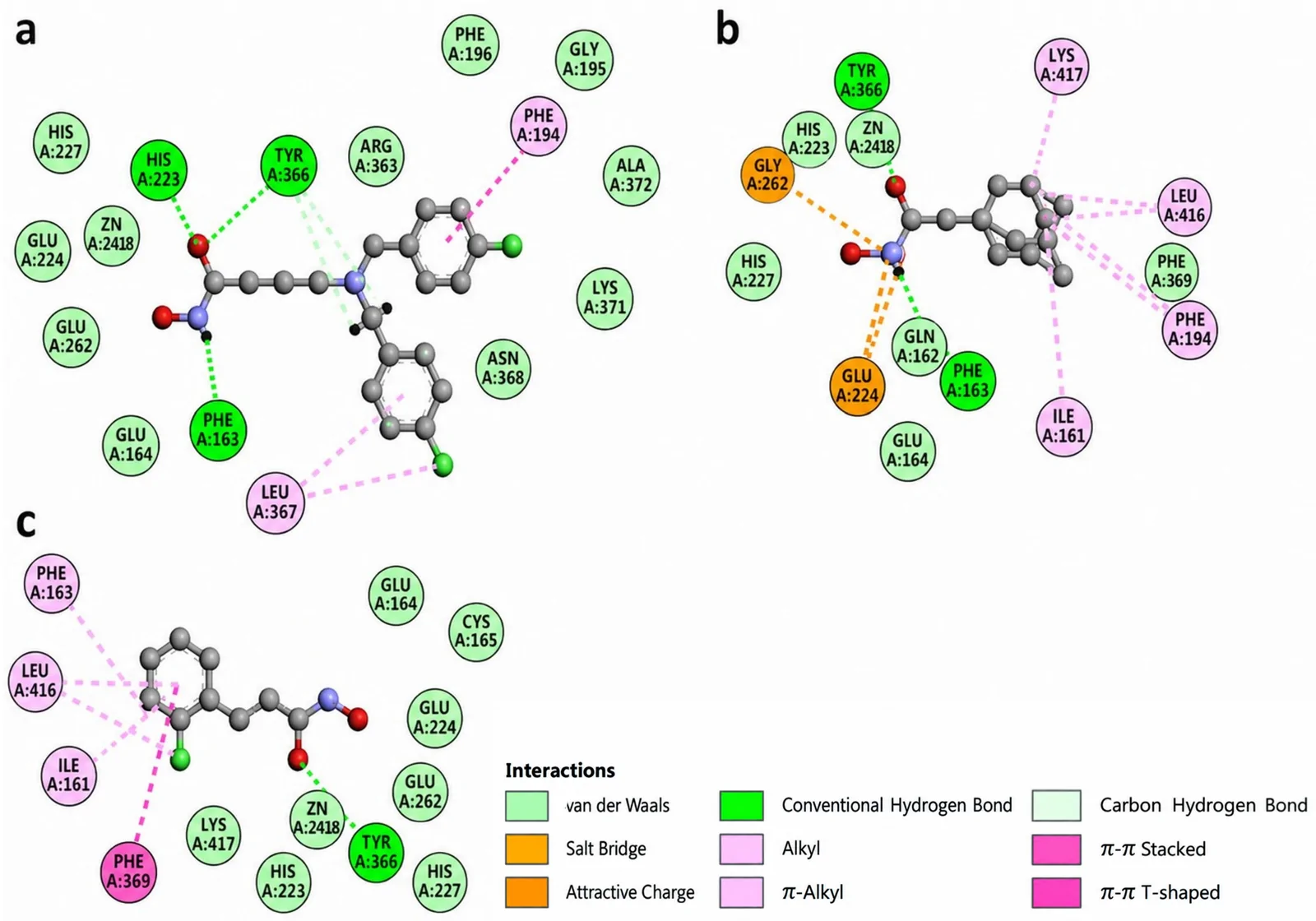
Intermolecular interactions in BoNT/A protein–ligand complexes from 100 ns MD simulations visualized using BIOVIA Discovery Studio software v21.1. Protein amino acid residues are represented as disks and ligands are shown as balls and sticks. Hydrophobic interactions are alkyl, π–alkyl, π–π stacked, and π–π T-shaped interactions, and electrostatic interactions are salt-bridge and attractive charge interactions. Labels for the residues partaking in the interactions are colored according to their respective type of interaction. (**a**) QI1-3QIY complex. (**b**) AXM-4HEV complex. (**c**) GB4-2IMA complex.

MD simulations were first carried out on the QI1-BoNT/A (3QIY), AXM-BoNT/A (4HEV), and GB4-BoNT/A (2IMA) complexes. These simulations served as a reference for probing hydrogen bonding and hydrophobic interactions between QI1, AXM, and GB4 inhibitors and their respective BoNT/A protein. Notably, the X-ray structures of these three inhibitors revealed specific interactions: H-bond interactions with residues Phe163, Glu224, and Tyr366, and hydrophobic interactions with residues Phe194, Tyr366, and Phe369. Figure 2 provides a visual representation of these interactions.

MD simulation results confirm that QI1 forms H-bond interactions with Phe163 and Tyr366, but also His223 and Arg363, as well as hydrophobic interactions with Leu367 and Phe194. Furthermore, van der Waals interactions were observed between QI1 and Glu164, Glu224, His227, Glu262, and the adjacent zinc ion to the hydroxamate moiety (Figure 2a). Analogous binding patterns were observed for AXM, where the hydroxamate moiety is surrounded by Glu164, His227, His223, and a zinc ion (Figure 2b). The inhibitor was further stabilized by H-bond interactions with Phe163 and Tyr366, electrostatic interactions with Glu224 and Glu262, and hydrophobic interactions with Ile161, Phe194, Leu416, and Lys417. GB4 was observed to form H-bond interactions with Tyr366, hydrophobic interactions with Ile161, Phe369, and Leu416, and van der Waals interactions with Phe163, Glu164, His223, Glu224, Glu262, and the adjacent zinc ion to the hydroxamate group (Figure 2c).

**Figure 3:**
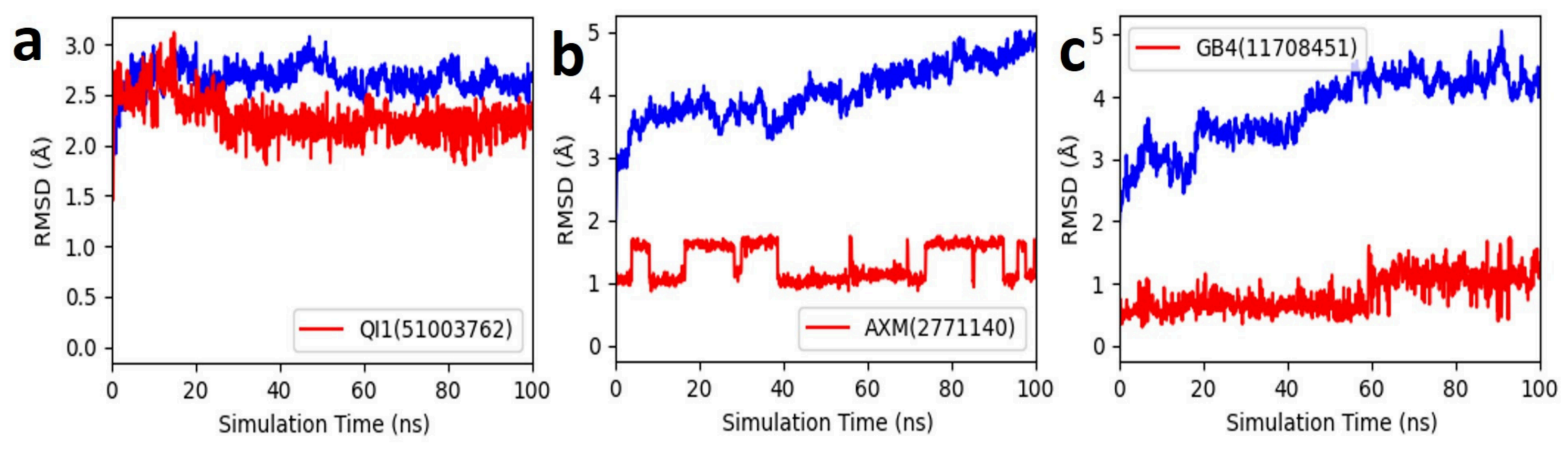
Root Mean Square Deviation (RMSD) of the protein backbone (blue) and ligand (red) from the 100 ns MD simulations of protein–ligand complexes. (**a**) QI1-3QIY (**b**) AXM-4HEV (**c**) GB4-2IMA.

The RMSD profiles of the protein backbone and ligands during the 100 ns MD simulations are shown in Figure 3 for the three co-crystallized ligand complexes. In each case, the protein backbone RMSD was calculated after least-squares fitting of the protein backbone to the energy-minimized starting structure, while the ligand RMSD was calculated after protein alignment to assess ligand positional stability within the binding pocket. For the QI1–3QIY, AXM–4HEV, and GB4–2IMA complexes, the average ligand RMSD values over the 100 ns trajectories were 2.2 Å, 1.3 Å, and 0.8 Å, respectively. The corresponding protein backbone RMSD profiles are shown in blue, while the ligand RMSD profiles are shown in red. The binding interaction trajectories of the 31 ligand complexes were further examined and used to support the binding free energy (BFE) analysis.

To further evaluate the stability and reproducibility of the highest-priority BoNT/A LC–ligand complexes, additional extended molecular dynamics simulations were performed for the five top-ranked candidates. The extended triplicate 300 ns MD simulations provided additional support for the stability of the five top-ranked BoNT/A LC–ligand complexes. Detailed plots from the three independent 300 ns replicate simulations of the five top-ranked complexes are provided in the Supplementary Information (Figures S4–S8). Across the replicate trajectories, the ligand RMSD profiles showed greater fluctuations than the protein backbone RMSD, reflecting expected ligand positional and conformational adjustments within the binding pocket; however, the absence of sustained increases in ligand RMSD, ligand–binding-site center-of-mass (COM) distance, and minimum protein–ligand distance indicated that the ligands generally remained associated with the BoNT/A LC binding region over the extended simulation timescale. Hydrogen-bond and contact analyses further supported the persistence of protein–ligand interactions during the simulations. The top-ranked ligands maintained dynamic hydrogen-bond interactions with the protein, and the hydrogen-bond donor–acceptor distance distributions were centered within geometrically reasonable ranges. In addition, key residue–ligand contact analysis showed that interactions with catalytically and structurally relevant residues were maintained across the replicate simulations, although the number and identity of individual contacts fluctuated over time. These extended replicate simulations strengthen the MD-based prioritization of the top five candidates while also highlighting the dynamic nature of ligand binding within the BoNT/A LC active site.

### 2.4. Binding Free Energies

**Figure 4:**
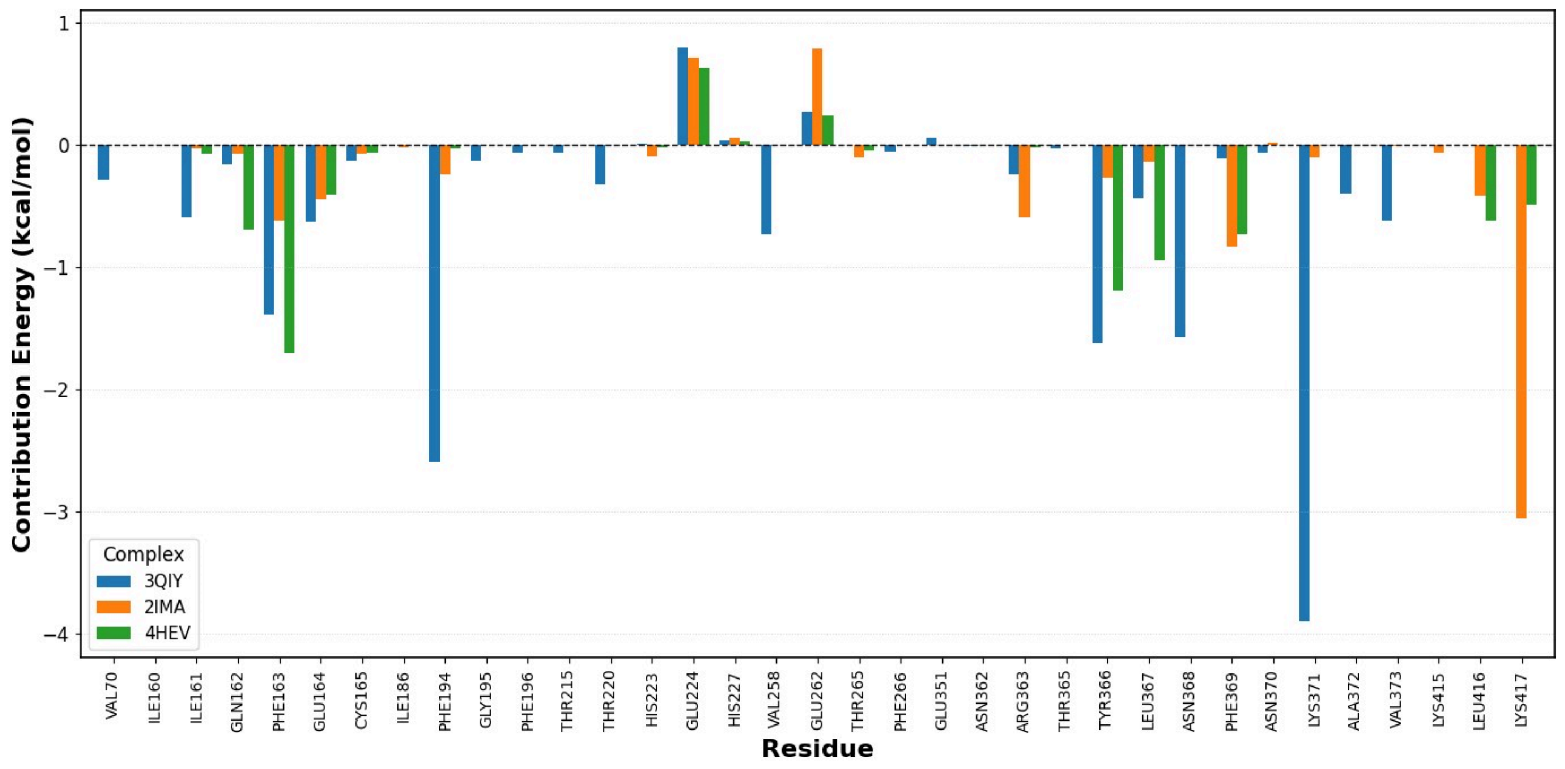
Contributions of individual residues with a high contribution to the total binding energy from MD simulations of the QI1-3QIY (blue), AXM-4HEV (green), and GB4-3QIY (orange) complexes.

MMGBSA-based BFE calculations were conducted on 31 selected protein–ligand complexes, followed by an assessment of individual residue energy contributions. For comparative analysis, the BFE and residue energy contributions were first evaluated for the three complexes QI1-BoNT/A (3QIY), AXM-BoNT/A (4HEV), and GB4-BoNT/A (2IMA). Ligands QI1, AXM, and GB4 exhibit BFE values of −36.84 kcal/mol, −28.47 kcal/mol, and −26.71 kcal/mol, respectively. The distribution of key residues’ contributions to the BFE is presented in Figure 4.

QI1 interacts strongly with residues Ile161, Phe163, Glu164, Phe194, Tyr366, Leu367, Asn368, and Lys371 of BoNT/A. The strong interactions with Phe194 and Leu367 may be attributed to hydrophobic interactions with QI1, while those observed for Phe163 and Tyr366 are due to H-bond interactions. Despite forming an H-bond with QI1, His223 contributed little to the total BFE. A potential explanation is that His223, part of the conserved HEXXH motif [27], is strongly coordinated to a zinc ion, rendering the H-bond interaction too weak (H-bond length of 2.64 Å compared to H-bond lengths of 1.90 Å and 2.07 Å observed in QI1-Phe163 and QI1-Tyr363, respectively; the reported bond lengths correspond to the hydrogen–acceptor distance rather than the donor–acceptor heavy-atom distance) to significantly contribute to the total BFE. The remaining residues likely derive their energy contribution from van der Waals interactions with QI1.

AXM interacts strongly with residues Ile161, Gln162, Phe163, Glu164, Phe194, Arg363, Tyr366, Phe369, Leu416, and Lys417. Like QI1, AXM features strong interactions with Phe163 and Tyr366, which could be attributed to H-bond interactions. Residue contributions to the BFE from Ile161, Phe194, Leu416, and Lys417 are due to hydrophobic interactions with AXM, while those for Gln162, Glu164, Arg363, and Phe369 involve van der Waals interactions.

**Figure 5:**
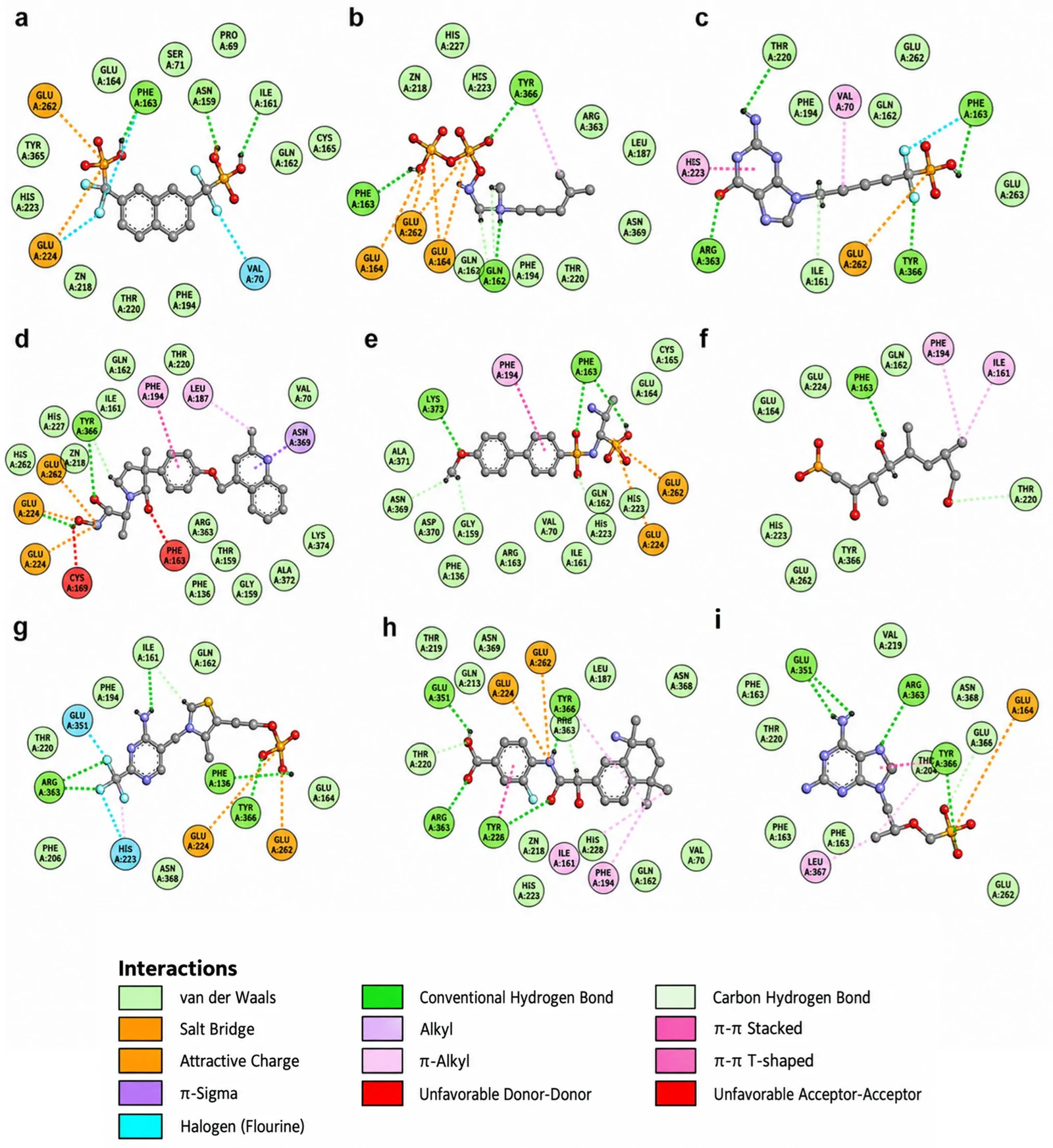
Binding interactions of potential inhibitors with BoNT/A LC. Protein amino acid residues are represented as disks and ligands are shown as balls and sticks. Intermolecular interactions are colored according to the type of interactions. (**a**) L09-3QIY (**b**) L07-3QIY (**c**) L25-3QIY (**d**) L31-3QIY (**e**) L20-3QIY (**f**) L14-3QIY (**g**) L30-3QIY (**h**) L24-3QIY and (**i**) L04-3QIY.

In the GB4-BoNT/A complex, residues Ile161, Phe163, Glu164, Phe194, Arg363, Tyr366, Phe369, Leu416, and Lys417 are involved in H-bond interactions, hydrophobic interactions, and van der Waals interactions, resulting in relatively high contributions to the BFE (Figure 5). Sixteen of the studied compounds exhibited predicted BFEs higher than that of QI1 (−36.84 kcal/mol), suggesting improved inhibitory activity against BoNT/A. The total predicted BFE values of these compounds are provided in Table 2, along with their van der Waals, electrostatic, polar solvation and non-polar separate contributions. The remaining compounds exhibited BFE values ranging from −36.17 kcal/mol to −8.25 kcal/mol (Supplementary Information Table S3), indicating moderate binding affinity with BoNT/A.

Residue energy contributions for the top nine protein–ligand complexes with the highest-magnitude MMGBSA-derived BFE values were analyzed in detail, as reported in Table 3. The energy decomposition analysis for the nine selected ligands reveals that residues Ile161, Gln162, Phe163, Glu164, Phe194, Thr220, His223, Glu224, His227, Glu262, Glu351, Arg363, Tyr366, Asn368, and Lys371 play crucial roles in ligand binding to BoNT/A. Interactions with these residues are mostly of the H-bond, electrostatic, hydrophobic, and van der Waals types.

## 3. Discussion

**Figure 6:**
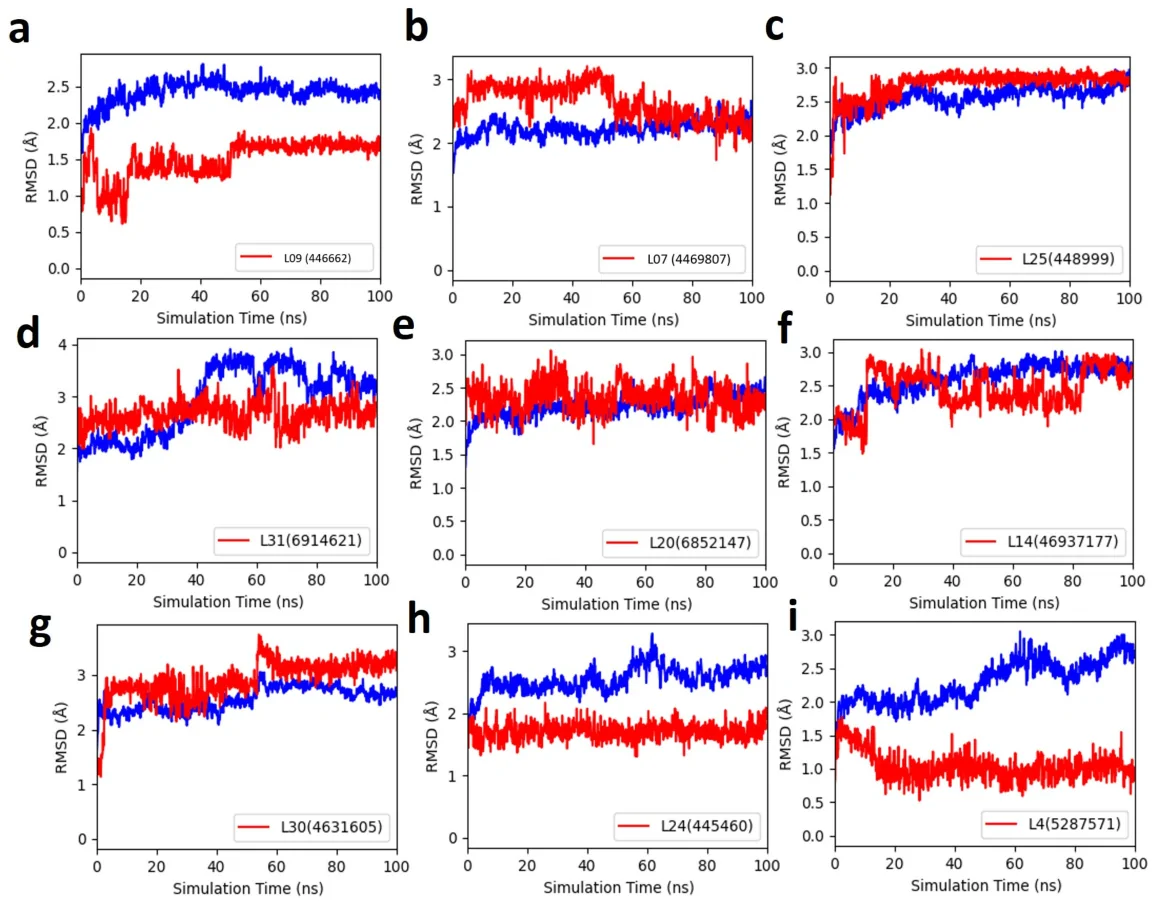
Root Mean Square Deviation (RMSD) of the protein backbone (blue) and ligand (red) from the 100 ns MD simulations of protein–ligand complexes. (**a**) L09-3QIY (**b**) L07-3QIY (**c**) L25-3QIY (**d**) L31-3QIY (**e**) L20-3QIY (**f**) L14-3QIY (**g**) L30-3QIY (**h**) L24-3QIY and (**i**) L04-3QIY.

In this study, we have identified nine ligands with high potential for BoNT/A inhibition based on their predicted total BFE and analyzed their structural features crucial for BoNT/A binding. The binding interactions and RMSD values of the complexes are depicted in Figure 5 and Figure 6, respectively. Overall, the results indicate that favorable binding to the BoNT/A LC active site is associated with a combination of hydrogen bonding, electrostatic interactions, hydrophobic contacts, fluorine-associated contacts, and van der Waals contributions involving key catalytic and binding-site residues.

The residue-level interaction and binding free energy decomposition analyses identified Ile161, Phe163, Glu164, Phe194, Thr220, His223, Glu224, Glu351, Arg363, Tyr366, and Asn368 as important contributors to ligand binding, in agreement with previous experimental and computational studies [9,10,16]. Interactions with Phe163, Arg363, and Tyr366 were particularly relevant because these residues are involved in stabilizing ligand binding within the catalytic pocket and have been implicated in the stabilization of the oxyanion generated during proteolytic cleavage [10,16,28]. Therefore, ligands containing hydrophobic and electronegative substituents capable of forming hydrogen-bond, electrostatic, or hydrophobic interactions with these residues may represent useful scaffolds for further evaluation as potential BoNT/A LC inhibitors. Such interaction patterns were observed for the reference inhibitors QI1 and GB4 (Figure 2), as well as for selected ligands identified in this study, including L04 and L30 (Figure 5).

Among the selected ligands, L09 showed the most favorable predicted BFE value of −70.46 kcal/mol. Its interaction profile included H-bond interactions with Ile161, Asp159, and Phe163; electrostatic interactions with Asp159, Glu224, and Glu262; fluorine-associated polar contacts with Val70, Phe163, and Glu224; and additional van der Waals interactions with surrounding active-site residues. The strong predicted binding of L09 appears to arise from the combined contribution of its aromatic scaffold, difluoromethyl group, and phosphonic acid moiety, which together support hydrophobic, electrostatic, and fluorine-associated polar contacts within the binding pocket. However, because this conclusion is based on computational modeling, L09 should be regarded as a high-priority candidate for experimental evaluation rather than as a confirmed inhibitor.

Other ligands with high-magnitude predicted BFE values also displayed extensive interaction networks with BoNT/A LC. L07 and L25, with BFE values of −57.27 and −57.14 kcal/mol, respectively, featured multiple H-bond interactions with active-site residues, together with hydrophobic and electrostatic contacts that likely contributed to their favorable predicted binding. L20 showed hydrogen-bond interactions involving several oxygen atoms with residues such as Gln162, Phe163, Gly195, Glu262, Asn368, and Lys371. L14, an aliphatic compound, formed hydrogen-bond interactions with Phe163 and Thr220, while L24 and L04 interacted through hydrogen bonds involving residues such as Phe163, Thr220, Glu351, Arg363, Glu164, and Tyr366. L30 formed H-bond interactions with Ile161, Phe163, Arg363, and Tyr366, as well as halogen-bond interactions involving His223 and Glu351.

A recurring feature among several prioritized ligands was the presence of phosphate, carboxylate, and fluorinated functional groups. L09, L07, L25, L20, L30, and L04 showed interaction patterns involving electrostatic and halogen-bond contributions associated with these groups. In particular, phosphate-containing ligands showed potential electrostatic interactions with residues such as Glu224 and Glu262, which contributed substantially to the predicted BFE decomposition profiles. These observations suggest that charged or highly polar substituents can contribute favorably to binding when properly oriented within the BoNT/A LC active site. However, such groups may also increase the risk of nonspecific or promiscuous binding to other metalloproteases, and their contribution should therefore be interpreted cautiously until selectivity studies are performed.

Fluorine-containing ligands were also observed to participate in interactions involving residues such as Phe163, Glu224, Glu351, Arg363, and Tyr366. These interactions may contribute to ligand stabilization through a combination of polar contacts, halogen-bond-like interactions, and hydrophobic effects. Halogen substituents can influence molecular recognition by improving shape complementarity, modifying local electrostatics, and supporting directional interactions with nucleophiles [29]. Halogen substituents that enable potentially favorable interactions within the catalytic pocket may induce conformational changes, as reported in previous experimental studies [9,10,30]. Nevertheless, the strength and specificity of such interactions depend strongly on the geometry of the ligand and the local protein environment. Therefore, the fluorinated ligands identified here should be considered as promising computational scaffolds requiring further biochemical and selectivity validation.

The hydroxamate-containing ligand L31 is particularly notable because hydroxamate groups are common zinc-binding motifs in several reported BoNT/A LC inhibitors. L31 formed H-bond interactions with Glu224 and Tyr366 and adopted an interaction pattern that partially resembles those of co-crystallized hydroxamate inhibitors. Reported BoNT/A LC inhibitors often contain zinc-binding groups such as hydroxamic acid [31,32], a thioacetyl group [33], or a hydroxyquinoline group [34]. However, some of these inhibitors are highly positively charged and lack a clearly identified zinc-binding group, suggesting an alternative mode of interaction with zinc ions. Further research is necessary to elucidate their mechanisms of action with zinc, which is beyond the scope of this study. In any case, the presence of a zinc-binding group may not be an absolute requirement for effective BoNT/A inhibition, as demonstrated by some of the top potential candidates uncovered in this work, although experimental validation would be required to confirm their inhibitory mechanisms.

The predicted BFE values of all top-ranked ligands, together with seven additional ligands listed in Table S3, were more favorable than those calculated for the co-crystallized reference inhibitors. These findings suggest that the identified ligands possess favorable computational binding profiles relative to the reference compounds. However, they should not be interpreted as experimentally confirmed BoNT/A LC inhibitors. Rather, the results support their prioritization for further biochemical testing, including SNAP-25 cleavage assays, selectivity profiling, and more detailed pharmacological evaluation. The known pharmacological backgrounds of some prioritized compounds also support their consideration in a repurposing context. For example, L09 is an aromatic compound containing fluoro and phosphonic acid moieties and has been reported to show moderate inhibitory activity against protein tyrosine phosphatase 1B [35]. L31 contains a hydroxamate moiety and has been reported as a potent and specific tumor necrosis factor alpha converting enzyme (TACE) inhibitor [36]. In addition, L26 (Supplementary Information Table S3), listed among the additional ligands in Table S3, is identified as dinoprost, a pharmaceutical agent formerly used to stimulate uterine muscle contractions during labor. These examples show that the screening workflow identified compounds from diverse pharmacological backgrounds, suggesting that their predicted BoNT/A LC binding is likely driven by local active-site chemical complementarity rather than global similarity between their original targets and BoNT/A LC.

The broader landscape of BoNT/A LC inhibitor research has recently been expanded with both repurposed small molecules and natural compound scaffolds identified via bioassay studies and computational docking [30,37,38], and emerging analyses suggest that polyphenol-like natural compounds may engage multiple domains of the neurotoxin in silico [37]. For example, KX2-361, an analog of the microtubule-disrupting agent tirbanibulin, was recently shown to inhibit BoNT/A LC activity using a docking-based approach [38], while flavonoid templates such as quercetagetin have been proposed as promising natural product-derived scaffolds [37]. In this context, the present virtual screening campaign expands the range of chemotypes predicted to interact favorably with the BoNT/A LC catalytic pocket. Importantly, however, successful BoNT/A inhibitor development requires more than favorable binding energetics. Candidate inhibitors must also demonstrate biochemical inhibition, selectivity over human metalloproteases such as neprilysin, angiotensin-converting enzyme, and matrix metalloproteases, cellular accessibility, and appropriate pharmacokinetic properties. In addition, intracellular trafficking and toxin persistence in neurons remain important challenges for the development of effective BoNT/A therapeutics [19].

A major limitation of the present study is that the findings are based entirely on computational analyses, including molecular docking, MD simulations, and MM/GBSA binding free energy calculations. Although additional 300 ns simulations were performed for the top five candidates to strengthen the assessment of protein–ligand stability, experimental validation remains essential. Therefore, the identified ligands should be regarded as computationally prioritized candidates for further evaluation rather than confirmed BoNT/A LC inhibitors. Future studies should include in vitro SNAP-25 cleavage assays, selectivity assessment against human metalloproteases, and further optimization of ligand potency, specificity, and cellular accessibility.

## 4. Materials and Methods

### 4.1. Binding Affinity Data Preparation and Validation

The 3D structures of BONT/A (PDB IDs: 2IMA, 3QIY, and 4HEV) were retrieved from the RCSB-PDB. Experimentally active (IC_50_ ≤ 5 μM) and inactive (IC_50_ > 5 μM) BoNT inhibitors were obtained from the BDB as a reference library for the purpose of validating our MF-based molecular docking protocol. Finally, the 3D structures of putative ligands were obtained from the DrugBank database.

The relative binding affinity (RBA) is the ratio of the binding strength exhibited by a reference compound (typically one with the highest established affinity) to that of the ligands of interest [39,40]. The conversion of the reference ligands’ IC_50_ values into empirical relative binding affinities (RBA) is simply obtained as

RBA = (IC_50_ of QI1)/(IC_50_ of ligand of interest) (1)

### 4.2. Estimation of QED Scores and Lipinski Rule of Five Drug-likeness Screening

We used a well-known approach, QED, introduced by Bickerton et al. [40], which assigns continuous scores indicative of drug-likeness. QED draws from eight attributes, encompassing factors like the number of H-bond acceptors (HBAs), number of H-bond donors (HBDs), molecular weight, partition coefficient (log P), molecular polar surface area (PSA), as well as number of rotatable bonds (ROTBs), aromatic rings (AROM), and structural alerts (ALERTS). The QED score is derived by calculating the geometric mean of desirability functions, each tailored to a distinct property. These functions are characterized by asymmetrical sigmoidal curves, fitted to the distribution of the corresponding physicochemical attributes of the ligand, and calibrated to reach a maximum value of 1. As such, the resulting QED score falls within the range of 0 to 1, with a higher QED score indicating a higher potential as a drug.

The molecular properties of 9137 ligands within the FDA-approved library dataset retrieved from the DrugBank [25] were calculated to assess their drug-likeness properties. The drug-likeness Screening Tool (DruLiTo, version 1.0), a Java-based open-source virtual screening application, was used to estimate different molecular and drug-likeness properties of the ligands. DruLiTo employs an array of drug-likeness rules, such as Lipinski’s rule [26], Veber’s rule [41], Ghose filter [42], BBB [43] and CMC-50-like rules [44]. With all rules initially applied to filter the dataset, only 1% of the ligands met the criteria. To increase the number of ligands for further analysis, we focused solely on Lipinski’s rule of five (Ro5) during initial screening. Of the 9137 ligands evaluated, 7576 complied with Ro5, while the remaining ligands, which violated the rule, were excluded from further analysis.

### 4.3. Molecular Docking-Based Virtual Screening

The VIRTUAL CHEMIST plugin of the MF platform [45] was employed to prepare ligand-protein structures and virtual screening. Using the CONVERT and SMART (Small Molecule Atom typing and Rotatable Torsion assignment) modules of MF, all the ligands in the dataset were protonated at physiological pH, and the energy was minimized with the AMBER force field. The protein structure was prepared using the PREPARE and ProCESS (Protein Conformational Ensemble System Setup) modules in MF. This process included the identification of missing atoms in incomplete residues of the crystal structures, modeling missing loop regions and residues, addition of hydrogen atoms, generation of probable tautomers, and assignment of bond orders and formal charges. A co-crystallized ligand was used to identify the active site. For each ligand, 100 docking poses were generated and scoring functions evaluated. Specifically, the “Score” mode in the FITTED (Flexibility Induced Through Targeted Evolutionary Description) module of MF was applied to virtual screening, and ligands were screened based on the Rank score. The top-rank scored ligands were selected for FITTED docking under “Dock” mode, and ligands were scrutinized based on the FITTED score and binding interactions. The same procedure was employed for the reference library of ligands from the BDB (Section 2.1) and the putative ligands from DrugBank (Section 2.2). Three different structures of BoNT/A (PDB IDs: 2IMA, 3QIY, and 4HEV) were used to investigate the binding interaction of ligands with the target. The results of docked BoNT/A-ligand complexes were visualized using the BIOVIA Discovery Studio 2021 visualizer [46].

### 4.4. MD Simulations

The 31 BoNT/A-ligand complexes with the best FITTED scores (i.e., highest-magnitude negative score) were selected for MD simulations. Each system was prepared using the CHARMM-GUI [47] interface with the CHARMM36m force field [48]. All simulations were performed with the GROMACS 2021 software package [49]. The TIP3P [50] explicit solvation water model was used. The system was neutralized using sodium chloride ions at a concentration of 0.15 M. Hydrogen mass repartitioning [51] was used so that a 4 fs time step integration could be used for all production runs. Equilibration was performed in the canonical (NVT) ensemble, while the isothermal–isobaric (NPT) ensemble was used for production. Through the 100 ns of MD production, the pressure was set at 1 atm using the Parrinello–Rahman barostat [52]. The temperature was set at 303.15 K using the V-rescale thermostat [53]. A distance cutoff of 12.0 Å was applied to short-range nonbonded interactions with a pair list distance of 12 Å, and Lennard–Jones interactions were smoothly truncated at 12 Å. Long-range electrostatic interactions were treated using the particle-mesh Ewald (PME) method [54], with a grid spacing of 1.0 Å for all simulation cells. All covalent bonds involving hydrogen atoms were constrained using the LINCS algorithm [55].

To further investigate the stability of the top-priority BoNT/A LC–ligand complexes, extended molecular dynamics simulations were carried out on the five leading candidates. Each selected complex had three independent 300 ns production MD runs, which resulted in 15 extended replicate trajectories overall. The replicate simulations were initiated using different initial velocity seeds while maintaining the same force field, ligand parameters, solvent model, temperature and pressure coupling schemes, and production simulation protocol used for the initial MD simulations. Analyses of the extended replicate simulations were conducted using protein backbone RMSD, ligand RMSD following protein alignment, root mean squared fluctuations (RMSFs), hydrogen-bond occupancy, hydrogen-bond distance distribution, ligand–binding-site center-of-mass (COM) distance, minimum protein–ligand distance, and key residue–ligand contact persistence. These analyses were used to assess ligand retention within the binding pocket, conformational stability of the complexes, flexibility of binding-site residues, and persistence of important protein–ligand interactions across independent trajectories.

### 4.5. Calculation of Binding Free Energy

The binding free energy (BFE or ΔG_bind_) of protein–ligand complexes was determined using the MMGBSA method [56] with the gmx_MMPBSA tool [57,58]. The MMGBSA approach computes the protein–ligand complex total BFE as well as the BFE for individual residues at the binding interface, using the MD simulation trajectory as input. This method relies on a thermodynamic cycle, wherein the BFE is separated into a molecular mechanics (MM) term in vacuum and polar/nonpolar solvation free energy terms [58]

ΔG_bind_ = ΔE_MM_ + ΔG_GB_ + ΔG_non-polar_ (2)

The polar solvation free energy is evaluated with the generalized Born model, and the nonpolar solvation free energy is given by an empirical expression that depends on the solvent-accessible surface area (SASA), within the MM/GBSA framework. For each protein–ligand complex, molecular configurations from 100 ns MD trajectories (1000 snapshots at regular time intervals) were used to evaluate the contributions of all amino acids located within 6 Å of the binding interface to the protein–ligand complex binding free energy.

## 5. Conclusions

In the quest for effective therapeutic agents against botulinum neurotoxin A (BoNT/A), we employed docking-based virtual screening to search a comprehensive compound library for potential BoNT/A inhibitors. Structure-based virtual screening, QED, MD simulations, and binding free energy calculations were conducted to investigate protein–ligand binding interactions and identify potential candidates for BoNT/A inhibition. Utilizing CADD methodologies, we identified 16 candidate ligands with binding affinities greater than those of the known BoNT/A inhibitors inside the co-crystallized structures. Detailed analysis of the binding interactions shows that hydrogen-bond, hydrophobic, van der Waals, and electrostatic interactions with key residues are vital for achieving high binding affinity of the inhibitors and stability of the resulting BoNT/A protein–ligand complexes. We recommend further experimental investigation of these potential inhibitors to validate their potential as effective BoNT/A inhibitors.

Moreover, the presence of halogen and functional groups, such as fluorine, carboxylic, and phosphate moieties, was found to enhance the binding affinity and specificity of the identified inhibitors. These findings identify additional functional moieties besides the zinc-chelating hydroxamate group common to most known BoNT/A inhibitors. Potent BoNT/A inhibitors can be designed by harnessing these structural features. The insights gained from this work should guide future efforts in the design and optimization of innovative therapeutic agents against BoNT/A-mediated pathologies.

## Figures and Tables

**Table 1:** Docking scores and FITTED energies (kcal/mol) of the top 16 selected ligands targeting BoNT/A LC ^a^.

Ligand (s)	PubChem ID	FITTED Score (FDS)	FITTED Energy
L09	446662	−61.7	−61.7
L07	4469807	−63.61	−102.52
L25	448999	−53.19	−75.5
L31	6914621	−52.71	−101.55
L20	6852147	−54.59	−80.92
L14	46937177	−55.72	−95.97
L30	4631605	−52.99	−76.81
L24	445460	−53.45	−67.93
L04	5287571	−68.17	−72.69
L18	9543420	−55.1	−65.79
L28	10342449	−53.07	−71.61
L26	5280363	−53.18	−82.84
L29	42609673	−53.01	−154.05
L22	23647361	−54.23	−99.97
L21	445455	−54.28	−77.56
L27	15942657	−53.1	−81.67
GB4	11708451	−38.58	−71.31
AXM	2771140	−49.05	−87.34
QI1	51003762	−42.52	−101.39

^a^ FDS—FITTED docking score; L—ligand from DrugBank library (numbers assigned to “L” do not follow any specific order); GB4—co-crystallized ligand in 2IMA; AXM—co-crystallized ligand in 4HEV; QI1—co-crystallized ligand in 3QIY used as references.

**Table 2:** BoNT/A LC protein–ligand BFE and intermolecular interaction contributions (kcal/mol) from MM-GBSA calculations ^a^.

Complex	Van der Waals	Electrostatics	Polar Solvation	Non-Polar	BFE
L09-3QIY	−27.64	−19.74	−17.77	−5.3	−70.46
L07-3QIY	−29.12	−17.99	−5.06	−5.10	−57.27
L25-3QIY	−30.82	−14.86	−6.10	−5.35	−57.14
L31-3QIY	−45.16	−7.73	3.66	−6.55	−55.77
L20-3QIY	−32.18	−12.88	−0.41	−5.63	−51.10
L14-3QIY	−21.52	−12.34	−10.85	−4.69	−49.40
L30-3QIY	−26.19	−22.52	4.62	−4.94	−49.03
L24-3QIY	−37.05	−9.56	3.40	−5.77	−48.99
L04-3QIY	−25.43	−14.30	−4.59	−4.46	−48.77
L18-3QIY	−34.99	−12.10	5.13	−5.80	−47.76
L28-3QIY	−41.06	−4.08	5.10	−6.20	−46.24
L26-3QIY	−28.82	−13.23	0.87	−4.85	−46.04
L29-3QIY	−39.13	−7.47	7.59	−6.18	−45.20
L22-3QIY	−34.81	−8.11	4.77	−6.21	−44.36
L21-3QIY	−34.01	−8.42	3.79	−5.54	−44.18
L27-3QIY	−34.23	−4.20	4.85	−5.09	−38.68
QI1-3QIY	−27.72	−5.19	2.02	−5.96	−36.84
AXM-3QIY	−19.09	−7.31	1.21	−3.28	−28.47
GB4-3QIY	−16.63	−8.12	0.99	−2.94	−26.71

^a^ Calculated using molecular configurations from 100 ns MD trajectories.

**Table 3:** Residue contributions to the BFE (kcal/mol) of selected BoNT/A 3QIY–ligand complexes ^a^.

Residue	L09	L07	L25	L31	L20	L14	L30	L24	L04
Val70	−2.33	−0.05	0.03	0.00	−1.33	−0.78	−0.28	−0.81	−0.37
Asp159	−4.93	−0.32	−0.19	−0.01	−0.63	−0.34	−0.41	−0.03	0.00
Ile161	−1.33	−0.19	−0.49	−0.43	−0.76	−1.09	−0.47	−0.55	−0.79
Gln162	−1.66	−0.15	−0.40	−0.62	−0.98	−2.56	−1.11	−0.20	−0.87
Phe163	−1.56	−2.66	−2.64	−1.18	−2.23	−2.94	−1.89	−2.66	−2.75
Glu164	−4.47	−6.25	−1.86	−0.74	−2.34	−2.20	−2.66	−1.23	−3.01
Cys165	−0.26	−0.25	−0.12	−0.07	−0.24	−0.17	−0.25	−0.10	−0.18
Phe194	−1.39	−2.92	−3.65	−3.24	−2.77	−2.35	−2.52	−2.54	−2.68
Thr215	0.03	−0.57	0.00	−0.30	−0.05	−0.04	−0.35	−0.25	−0.15
Thr220	−0.31	−1.43	−0.90	−1.03	−0.36	−0.57	−0.47	−1.24	−1.44
His223	−1.75	−1.97	−1.82	−0.79	−1.19	−1.01	−1.28	−1.06	−2.10
Glu224	−4.34	−3.97	−3.80	0.45	−3.92	−3.29	−3.81	0.59	−3.04
His227	−0.74	−0.72	−0.70	0.02	−0.59	−0.55	−0.64	0.03	−0.67
Glu262	−5.23	−5.06	−5.41	0.21	−3.84	−2.66	−3.88	0.43	−4.39
Glu351	−0.91	−1.36	−0.84	−0.06	0.00	0.00	−0.38	0.42	−0.75
Arg363	0.25	−1.54	−2.52	−1.95	0.12	−0.06	−1.09	−0.73	−0.35
Tyr366	−0.16	−0.31	−0.51	−2.73	−0.24	−0.28	−0.28	−0.56	−0.27
Asn368	−0.37	−0.44	−0.85	−1.19	−0.97	−0.07	−1.18	−0.22	−0.30
Lys371	0.63	0.00	0.00	−2.04	−1.36	−0.13	0.00	−1.01	0.00
Val373	−0.08	0.00	0.00	−0.55	−0.44	−0.34	0.00	−0.28	0.00

^a^ Obtained from MMGBSA calculations. Only amino acids located within 6 Å of the binding interface are considered. See Figure 5 for details of the interactions.

## Data Availability

All structural and simulation data are available upon request (email). All remaining data are contained within the manuscript.

## Supplementary Materials

The following supporting information can be downloaded at: https://www.mdpi.com/article/10.3390/ijms27188081/s1.
