# Supplementary material for: Unveiling Novel Metalloprotease Inhibitors Targeting Botulinum Neurotoxin Through Structure-Based Virtual Screening of Drug Libraries and Molecular Dynamics Simulations
Source: Int J Mol Sci. 2026 Sep 11;27(18):8081. doi: 10.3390/ijms27188081 (PMC13607544; doi:10.3390/ijms27188081)
Supplement: Supplementary file 1 [file ijms-27-08081-s001.zip › ijms-4207414-supplementary.pdf]

Supplementary Information

Unveiling Novel Metalloprotease Inhibitors Targeting Botulinum Neurotoxin through Structure-Based Virtual Screening of Drug Libraries and Molecular Dynamics Simulations

Ridwan Sulaimon,[1] Gurudeeban Selvaraj,[1] Nora W.C. Chan,[2] Anguang Hu[2] and Gilles H. Peslherbe[1]

[1] *Centre for Research in Molecular Modeling and Department of Chemistry and Biochemistry, Concordia University, Montreal, QC, H4B 1R6, Canada*

[2] *Suffield Research Centre, Defence Research and Development Canada, Medicine Hat, AB, T1A 8K6, Canada*

List of tables

Table S1. Estimated logRBA values of reference ligands with BoNT/A LC. 2
Table S2. Selected compounds PubChem ID and FITTED docking score 3
Table S3. BoNT/A LC protein-ligand binding free energy (ΔG_{bind}) 4

List of Figures

Figure S1. Intermolecular interactions in BoNT/A LC (3QIY) and QI1 complexes 5
Figure S2. Intermolecular interactions in BoNT/A LC (4HEV) and AXM complexes 6
Figure S3. Intermolecular interactions in BoNT/A LC (2IMA) and GB4 complexes 7
Figure S3. Extended MD trajectory analyses for the L09–BoNT/A LC complex 8
Figure S5. Extended MD trajectory analyses for the L07–BoNT/A LC complex 9
Figure S6. Extended MD trajectory analyses for the L20–BoNT/A LC complex 10
Figure S7. Extended MD trajectory analyses for the L25–BoNT/A LC complex 11
Figure S8. Extended MD trajectory analyses for the L31–BoNT/A LC complex 12
Bibliography 13

Table S1. Estimated logRBA values of reference ligands with BoNT/A LC.[a]

BDB ID	Ref ID	Type	RBA	logRBA	FITTED Score
42387	BDB1	Active	7.667	0.885	-38.26
42382	BDB2	Active	6.571	0.818	-41.17
42386	BDB3	Active	5.750	0.760	-42.67
42374	BDB4	Active	5.111	0.709	-35.56
42389	BDB5	Active	2.300	0.362	-36.54
52651	BDB6	Active	1.917	0.283	-36.22
52644	BDB7	Active	1.586	0.200	-19.98
42371	BDB8	Active	1.484	0.171	-29.27
42396	BDB9	Active	1.438	0.158	-21.83
42381	BDB10	Active	1.045	0.019	-34.29
52637	BDB11	Active	0.958	-0.018	-20.24
42384	BDB12	Active	0.902	-0.045	-35.76
52548	BDB13	Active	0.844	-0.074	-20.54
52648	BDB14	Active	0.821	-0.085	-19.98
52642	BDB15	Inactive	0.697	-0.157	-22.61
44811	BDB16	Inactive	0.657	-0.182	-21.36
42395	BDB17	Inactive	0.657	-0.182	-23.13
52649	BDB18	Inactive	0.639	-0.195	-22.36
42356	BDB19	Inactive	0.605	-0.218	-23.56
52547	BDB20	Inactive	0.562	-0.250	-24.05
42383	BDB21	Inactive	0.511	-0.291	-36.43
52492	BDB22	Inactive	0.473	-0.325	-22.14
44809	BDB23	Inactive	0.460	-0.337	-15.34
42393	BDB24	Inactive	0.460	-0.337	-17.30
52643	BDB25	Inactive	0.460	-0.337	-22.41
52494	BDB26	Inactive	0.414	-0.383	-22.55
52487	BDB27	Inactive	0.390	-0.409	-19.20
44815	BDB28	Inactive	0.390	-0.409	-21.98
42391	BDB29	Inactive	0.383	-0.416	-35.56
	AXM	Active	4.600	0.663	-49.05
	QI1	Active	1.000	0.000	-42.52
	GB4	Active	11.220	1.050	-35.57

[a]Reference ligands are obtained from Binding DB(BDB) library; BDB- ligand from Binding Database; RBA-relative binding affinity.

Table S2. Selected compounds PubChem ID and FITTED docking score.[a]

Sl. No	PubChem ID	FITTED Score	Sl. No	PubChem ID	FITTED Score	Sl. No	PubChem ID	FITTED Score
1	5459816	-98.21	32	445455	-54.28	63	46937150	-51.55
2	5287714	-85.34	33	23647361	-54.23	64	9935681	-51.53
3	4469994	-83.79	34	10172821	-53.93	65	23633673	-51.49
4	9084	-83.17	35	445460	-53.45	66	6918638	-51.46
5	5288817	-80.09	36	448999	-53.19	67	5311236	-51.43
6	44	-77.70	37	5280363	-53.18	68	447110	-51.37
7	200164	-73.75	38	15942657	-53.10	69	446905	-51.35
8	16133778	-71.39	39	10342449	-53.07	70	5289105	-51.31
9	5288136	-70.30	40	42609673	-53.01	71	6540261	-51.28
10	5460580	-69.91	41	4631605	-52.99	72	11256664	-51.27
11	5287571	-68.17	42	5289457	-52.93	73	3256857	-51.11
12	3396559	-67.64	43	6313595	-52.82	74	24946280	-51.10
13	5288442	-66.52	44	6419228	-52.77	75	735	-50.97
14	4437	-65.56	45	6914621	-52.71	76	25052023	-50.82
15	4469807	-63.61	46	23582412	-52.70	77	5289389	-50.56
16	5287837	-62.85	47	46911863	-52.69	78	11242315	-50.51
17	446662	-61.70	48	9547915	-52.66	79	10379137	-50.48
18	123982	-59.30	49	5111	-52.57	80	5279172	-50.45
19	5287517	-57.15	50	446665	-52.50	81	5289215	-50.35
20	1003	-57.09	51	5312125	-52.32	82	766948	-50.35
21	24808499	-57.04	52	66440	-52.22	83	5362	-50.28
22	175	-56.40	53	5280723	-52.12	84	33675	-50.28
23	5287492	-56.04	54	446056	-52.11	85	60195662	-50.24
24	46937177	-55.72	55	5276507	-51.90	86	24795088	-50.24
25	5289593	-55.60	56	448087	-51.89	87	11143173	-50.23
26	157920	-55.43	57	44400013	-51.78	88	1290	-50.20
27	9820008	-55.39	58	24178109	-51.78	89	35375	-50.16
28	9543420	-55.10	59	5180212	-51.72	90	10430040	-50.10
29	59422203	-54.92	60	5288561	-51.67	91	24771824	-50.06
30	5289158	-54.73	61	3256861	-51.58	92	466151	-50.01
31	6852147	-54.59	62	6102762	-51.55			

[a]Evaluated with Molecular Forecaster.

Table S3. BoNT/A LC protein-ligand binding free energy (ΔG_{bind}) and intermolecular interaction contributions (kcal/mol) from MMGBSA calculations for all 31 ligands investigated.[a]

Complex	PubChem ID	Van der Waals	Electrostatics	Polar solvation	Non-polar	ΔG_{bind}
L16-3QIY	157920	-33.02	-2.73	4.92	-5.34	-36.17
L08-3QIY	5287837	-16.30	-11.47	-4.71	-3.58	-36.05
L11-3QIY	5287517	-11.63	-14.93	-3.65	-3.36	-33.58
L10-3QIY	123982	-22.13	-7.23	2.99	-4.18	-30.55
L17-3QIY	9820008	-27.61	-1.51	3.58	-3.75	-29.30
L12-3QIY	24808499	-34.11	-52.22	62.58	-4.45	-28.20
L13-3QIY	5287492	-28.30	-22.43	28.10	-4.73	-27.37
L06-3QIY	4437	-12.72	-25.43	18.03	-3.26	-23.38
L19-3QIY	59422203	-22.00	-2.13	4.44	-3.25	-22.94
L23-3QIY	10172821	-21.66	-24.92	31.34	-3.98	-19.22
L03-3QIY	5288136	-12.91	-8.36	7.39	-3.44	-17.32
L05-3QIY	5288442	-11.22	-3.81	5.00	-2.39	-12.41
L01-3QIY	5287714	-9.03	-2.73	4.17	-1.89	-9.48
L02-3QIY	4469994	-5.30	-1.40	-1.18	-0.91	-8.79
L15-3QIY	5289593	-7.22	-2.99	3.40	-1.44	-8.25

[a]Calculated using the last 1000 snapshots of 100-ns MD trajectories.

a

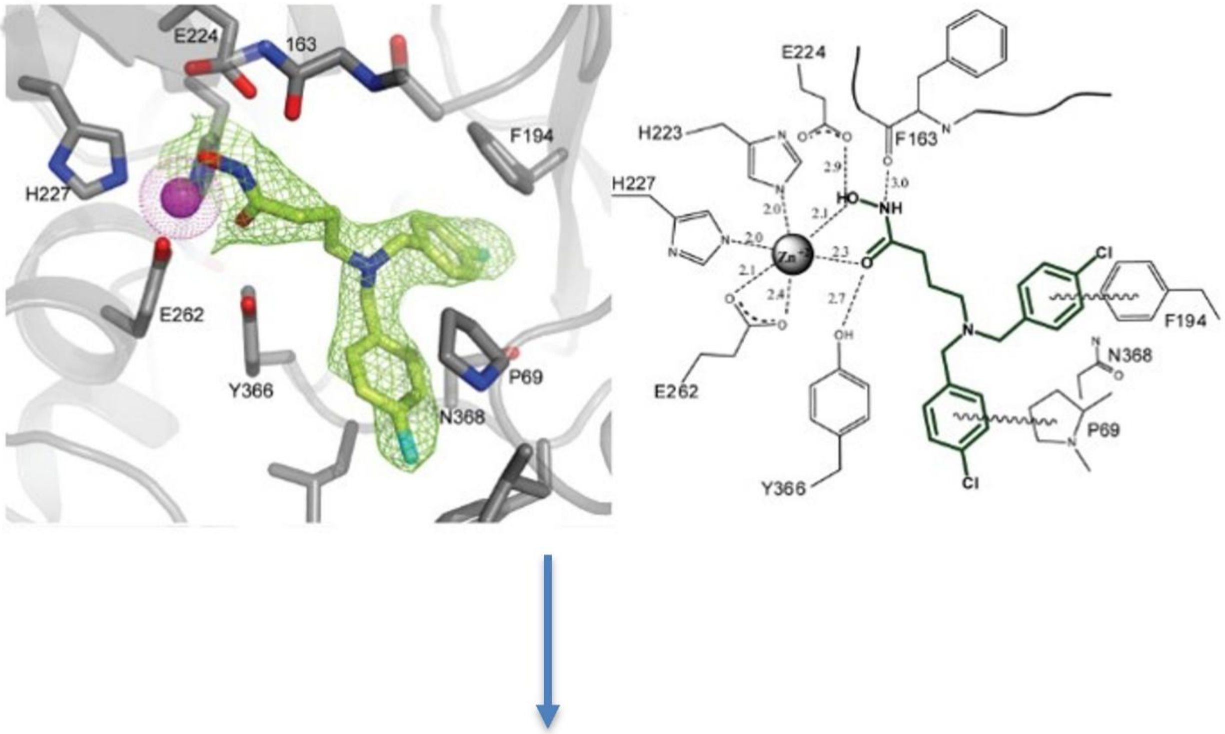

b

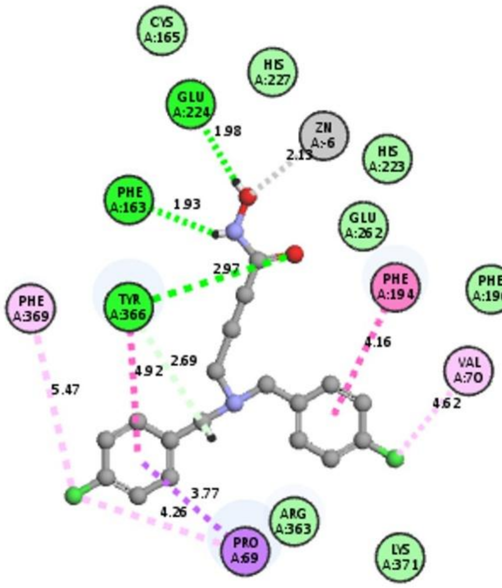

Figure S1. Intermolecular interactions in BoNT/A LC (3QIY) and QI1 complexes. a) The crystal structure of the complex [1]. (b) Binding interactions of the complex obtained from molecular docking studies.

a

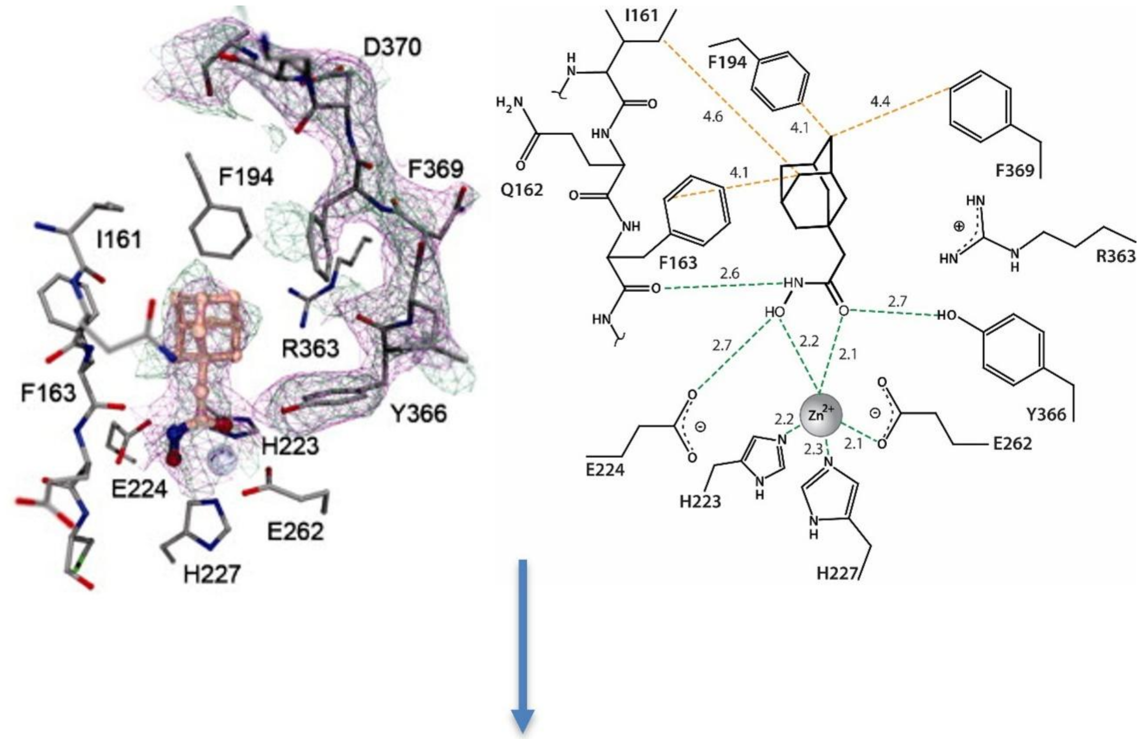

b

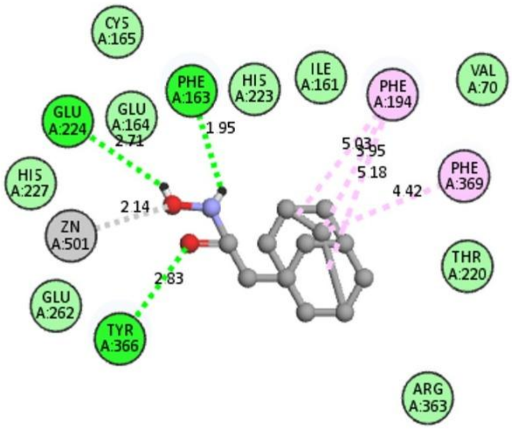

Figure S2. Intermolecular interactions in BoNT/A LC (4HEV) and AXM complexes. a) The crystal structure of the complex [2]. (b) Binding interactions of the complex obtained from molecular docking studies.

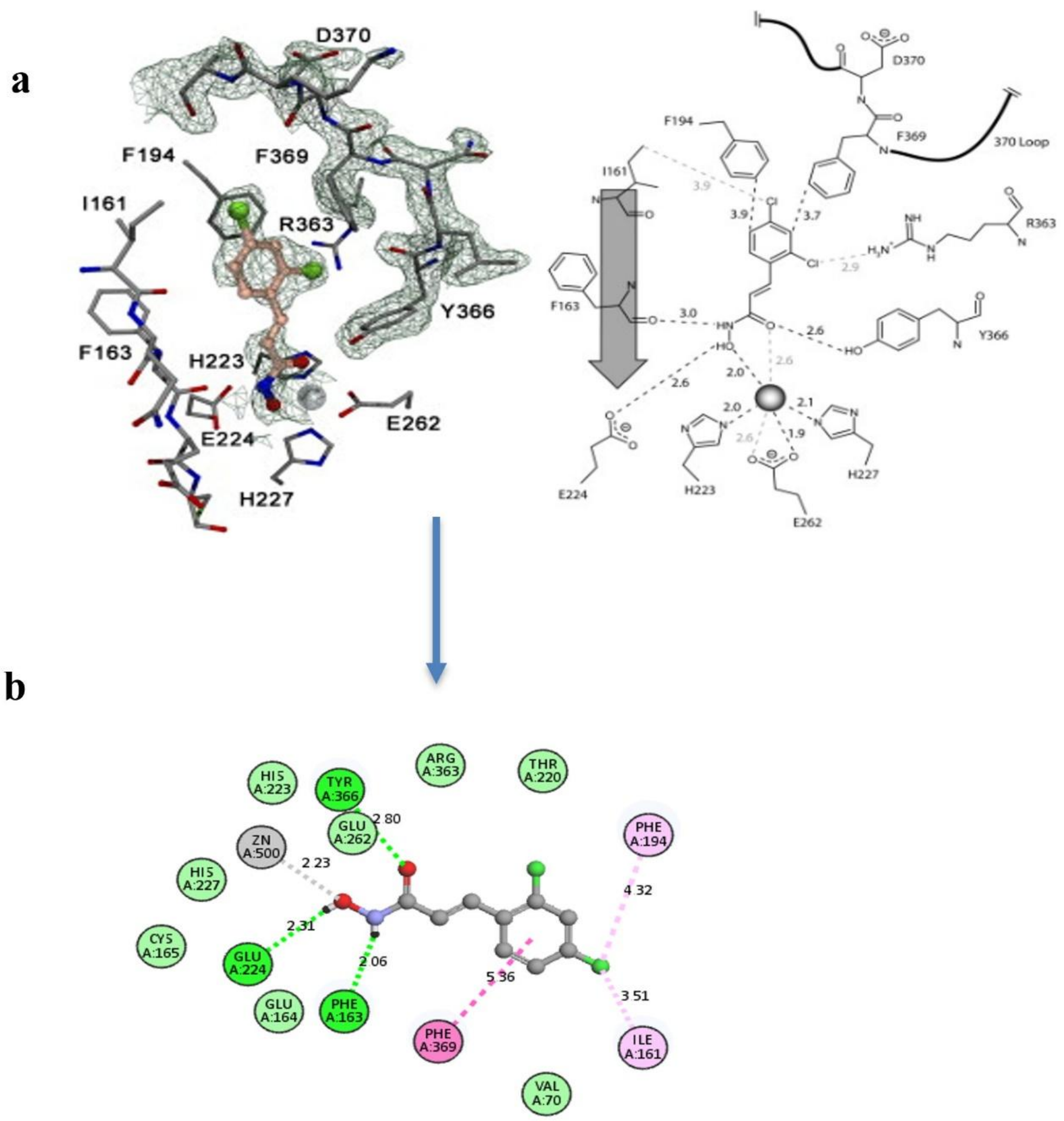

Figure S3. Intermolecular interactions in BoNT/A LC (2IMA) and GB4 complexes. a) The crystal structure of the complex [3]. (b) Binding interactions of the complex obtained from molecular docking studies.

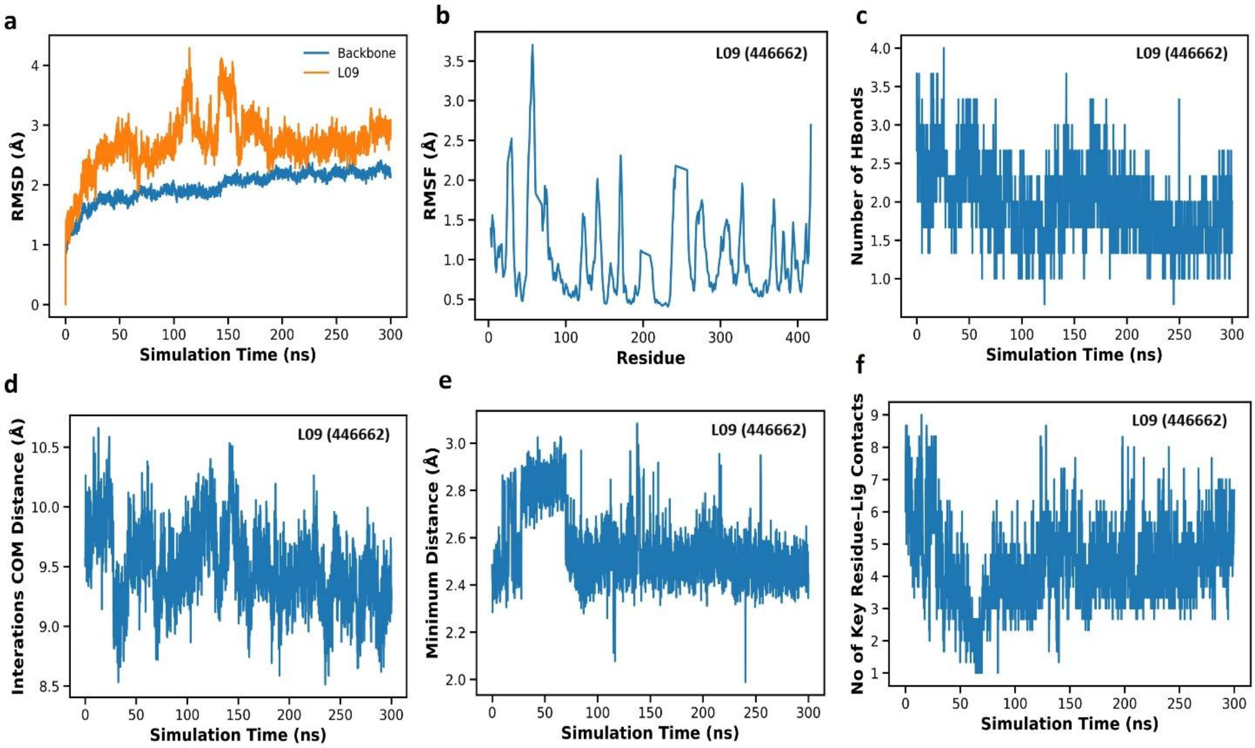

Figure S4. Extended MD trajectory analyses for the L09–BoNT/A LC complex. Replicate-averaged analyses from three independent 300 ns MD simulations of the L09–BoNT/A LC complex. (a) RMSD profiles of the protein backbone and L09. (b) Residue-based RMSF profile of BoNT/A LC in complex with L09. (c) Number of protein–ligand hydrogen bonds formed during the simulations. (d) Ligand–binding-site center-of-mass distance, used to monitor retention of L09 within the binding pocket. (e) Minimum protein–ligand distance throughout the simulations. (f) Number of persistent contacts between L09 and key BoNT/A LC binding-site residues.

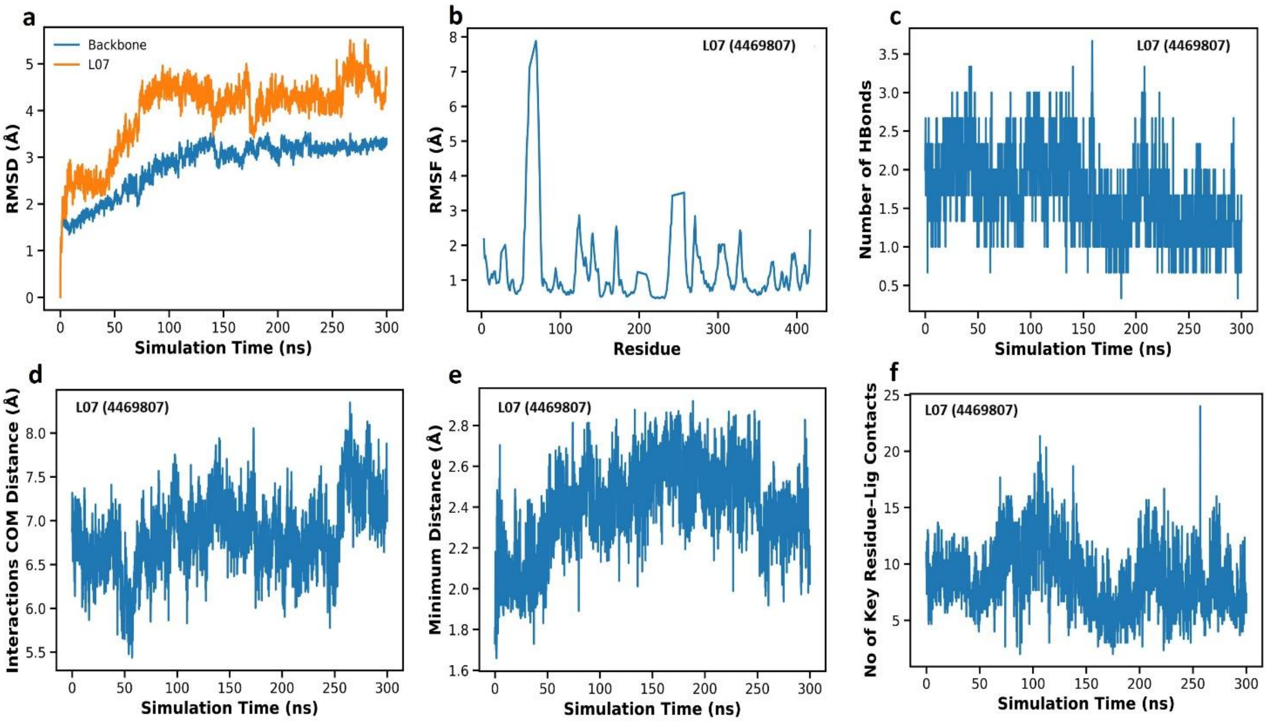

Figure S5. Extended MD trajectory analyses for the L07–BoNT/A LC complex. Replicate-averaged analyses from three independent 300 ns MD simulations of the L07–BoNT/A LC complex. (a) RMSD profiles of the protein backbone and L07. (b) Residue-based RMSF profile of BoNT/A LC in complex with L07. (c) Number of protein–ligand hydrogen bonds formed during the simulations. (d) Ligand–binding-site center-of-mass distance, used to monitor retention of L07 within the binding pocket. (e) Minimum protein–ligand distance throughout the simulations. (f) Number of persistent contacts between L07 and key BoNT/A LC binding-site residues.

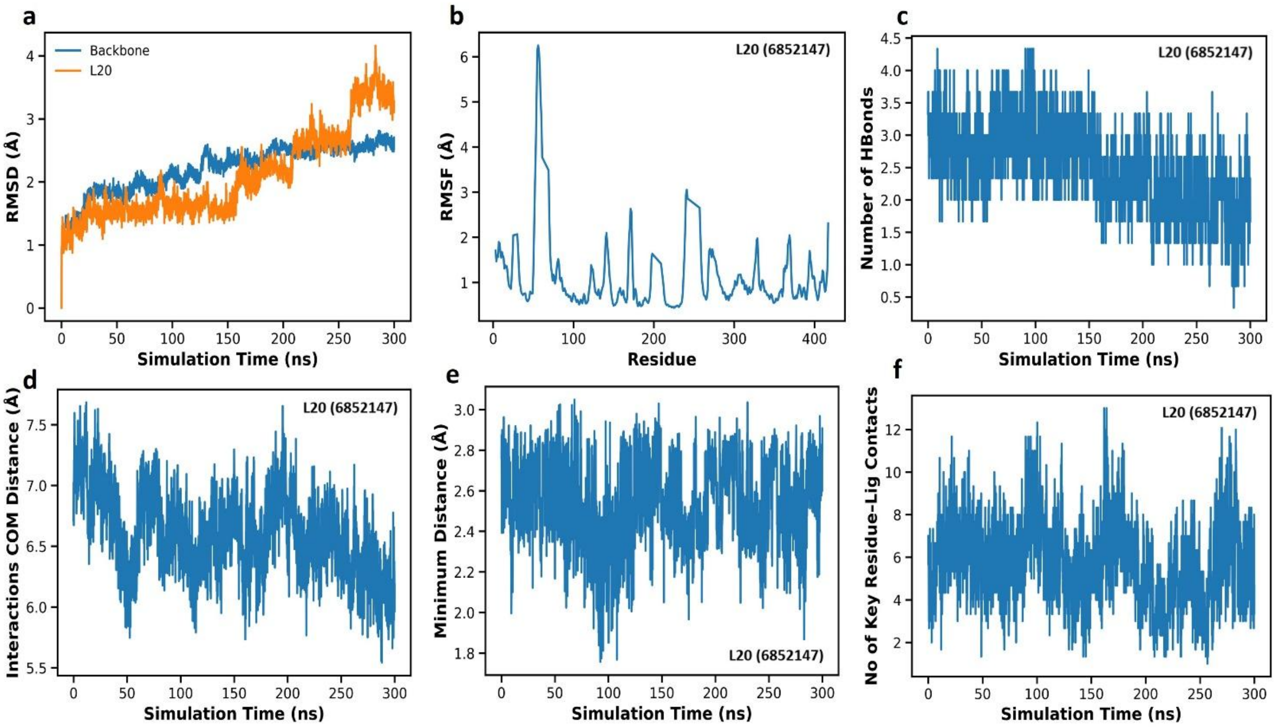

Figure S6. Extended MD trajectory analyses for the L20–BoNT/A LC complex. Replicate-averaged analyses from three independent 300 ns MD simulations of the L20–BoNT/A LC complex. (a) RMSD profiles of the protein backbone and L20. (b) Residue-based RMSF profile of BoNT/A LC in complex with L20. (c) Number of protein–ligand hydrogen bonds formed during the simulations. (d) Ligand–binding-site center-of-mass distance, used to monitor retention of L20 within the binding pocket. (e) Minimum protein–ligand distance throughout the simulations. (f) Number of persistent contacts between L20 and key BoNT/A LC binding-site residues.

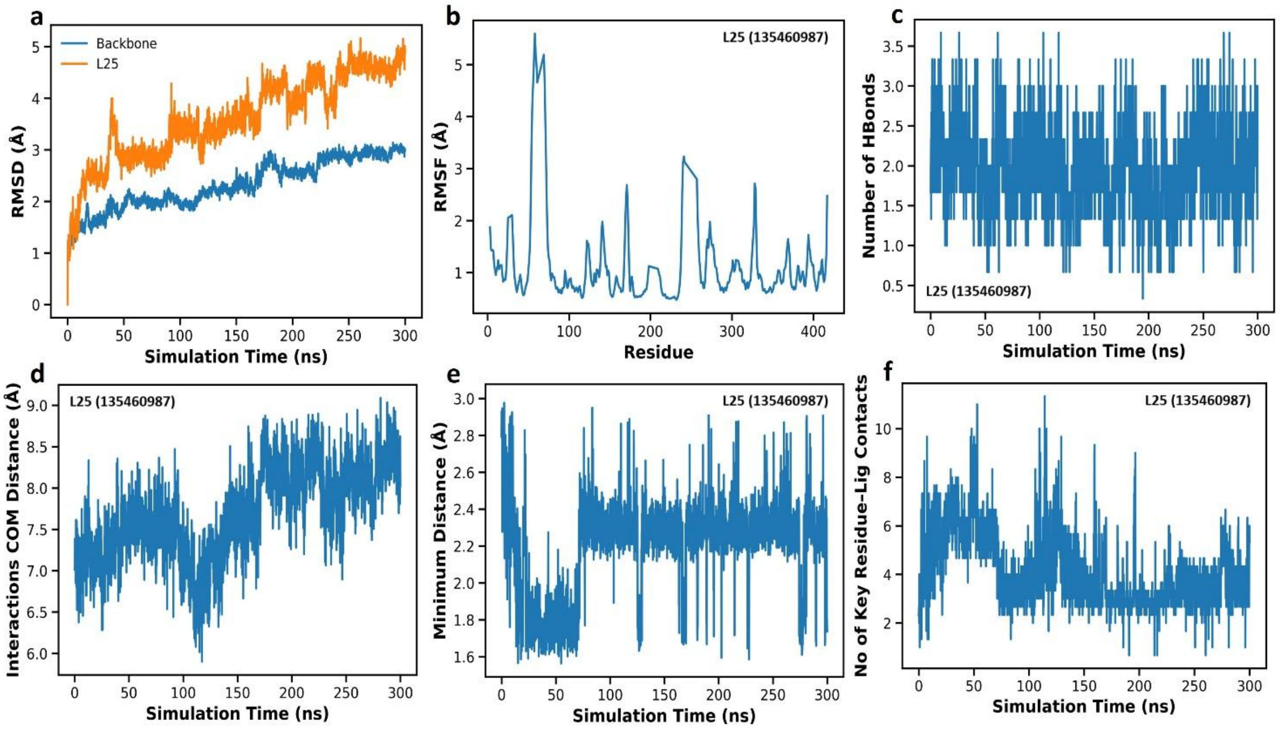

Figure S7. Extended MD trajectory analyses for the L25–BoNT/A LC complex. Replicate-averaged analyses from three independent 300 ns MD simulations of the L25–BoNT/A LC complex. (a) RMSD profiles of the protein backbone and L25. (b) Residue-based RMSF profile of BoNT/A LC in complex with L25. (c) Number of protein–ligand hydrogen bonds formed during the simulations. (d) Ligand–binding-site center-of-mass distance, used to monitor retention of L25 within the binding pocket. (e) Minimum protein–ligand distance throughout the simulations. (f) Number of persistent contacts between L25 and key BoNT/A LC binding-site residues.

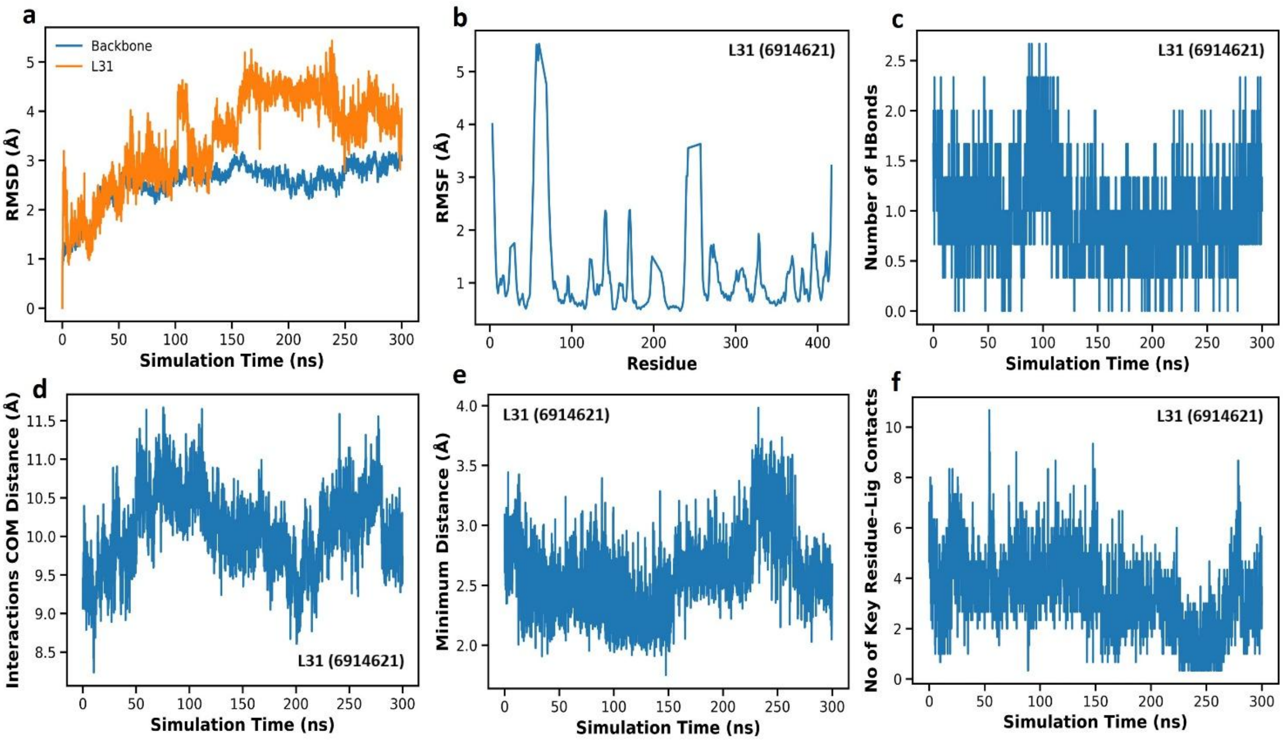

Figure S8. Extended MD trajectory analyses for the L31–BoNT/A LC complex. Replicate-averaged analyses from three independent 300 ns MD simulations of the L31–BoNT/A LC complex. (a) RMSD profiles of the protein backbone and L31. (b) Residue-based RMSF profile of BoNT/A LC in complex with L31. (c) Number of protein–ligand hydrogen bonds formed during the simulations. (d) Ligand–binding-site center-of-mass distance, used to monitor retention of L31 within the binding pocket. (e) Minimum protein–ligand distance throughout the simulations. (f) Number of persistent contacts between L31 and key BoNT/A LC binding-site residues.

Bibliography

[1] A. A. Thompson *et al.*, "Structural characterization of three novel hydroxamate-based zinc chelating inhibitors of the Clostridium botulinum serotype a neurotoxin light chain metalloprotease reveals a compact binding site resulting from 60/70 loop flexibility," *Biochemistry*, vol. 50, no. 19, pp. 4019–4028, May 2011, doi: 10.1021/bi2001483.

[2] P. Šilhár *et al.*, "Evaluation of adamantane hydroxamates as botulinum neurotoxin inhibitors: Synthesis, crystallography, modeling, kinetic and cellular based studies," *Bioorg Med Chem*, vol. 21, no. 5, pp. 1344–1348, Mar. 2013, doi: 10.1016/j.bmc.2012.12.001.

[3] N. R. Silvaggi *et al.*, "Structures of clostridium botulinum neurotoxin serotype a light chain complexed with small-molecule inhibitors highlight active-site flexibility," *Chem Biol*, vol. 14, no. 5, pp. 533–542, May 2007, doi: 10.1016/j.chembiol.2007.03.014.
